# Alternative approaches to phase III clinical trials for vaccine efficacy and licensure: the role of real-world evidence – a meeting report

**DOI:** 10.1016/j.biologicals.2026.101886

**Published:** 2026-08

**Authors:** Laurence de Moerlooze, Alexander Allen, Ilaria Bartalesi, Marco Cavaleri, Miles P. Davenport, Bradford D. Gessner, Brenda Gomes, Kyla Hayford, Alane Izu, Hector S. Izurieta, Victoria A. Jenkins, Randee Kastner, Phil Krause, Joshua Nealon, Lidia Oostvogels, Arto A. Palmu, Robert C. Read, Carla Saenz, Dean K. Smith, Suely Tuboi, Frank Vandendriessche, Marc Baay, Danielle Craig, Pieter Neels, Kaatje Bollaert

**Affiliations:** a P95, Leuven, Belgium; b Field Epidemiology and Health Protection Units, Public Health England, London, UK; c GSK, Siena, Italy; d EMA, Amsterdam, Netherlands; e Infection Analytics Program, Kirby Institute, UNSW Sydney, Kensington, NSW, 2052, Australia; f EpiVac Consulting, Anchorage, AK, USA; g Brazilian Health Regulatory Agency (ANVISA), Brasilia, Brazil; h Vaccine Medical Affairs, Pfizer, Inc., New York, NY, USA; i South African Medical Research Council Vaccines and Infectious Diseases Analytics Research Unit, Faculty of Health Science, University of the Witwatersrand, Johannesburg, South Africa; j Office of Vaccines Research and Review, Center for Biologics Evaluation and Research, US FDA, Silver Spring, MD, USA; k Bavarian Nordic Belgium, Brussels, Belgium; l Takeda Pharmaceuticals International AG, Zurich, Switzerland; m Bethesda, MD, USA; n Sanofi, Lyon, France; o MinervaX ApS, Frederiksberg, Denmark; p FVR – Finnish Vaccine Research, Tampere, Finland; q NIHR Southampton Biomedical Research Centre, University Hospital Southampton NHS Foundation Trust, Southampton, UK; r Faculty of Medicine and Institute for Life Sciences, University of Southampton, Southampton, UK; s Department of Evidence and Intelligence for Action in Health, Pan American Health Organization, Washington, DC, USA; t IABS North America, Canada; u Takeda Pharmaceuticals Brazil, São Paulo, Brazil; v Ficaja Farma, Huldenberg, Belgium; w Coalition for Epidemic Preparedness Innovations, P.O. BOX 123, Torshov, 0412, Oslo, Norway; x IABS, Geneva, Switzerland

## Abstract

• Alternative methods for vaccine licensure are needed for situations when phase III clinical trials are not feasible.
• From emergency use to full approval, integration of RWE alongside traditional data sources needs to be promoted.
• Safe, ethically robust human infection models can generate insights into transmission biology, CoPs, and vaccine efficacy.
• Pragmatic randomised trials can offer a balance between methodological rigour and real-world applicability.
• Post-licensure pragmatic trials offer a regulatory pathway for confirming and/or expanding pre-licensure data.

## Abbreviations

CHIMs -: Controlled human infection models
CoPs -: Correlates of protection
DRC –: Democratic Republic Congo
US FDA -: United States Food & Drug Administration
GBS -: Group B Streptococcus
IAP -: Intrapartum antibiotic prophylaxis
IPD –: Invasive pneumococcal disease
LRTI –: Lower respiratory tract infection
OPA -: Opsonophagocytic activity
PCVs -: Pneumococcal conjugate vaccines
RCTs -: Randomised controlled trials
RSV -: Respiratory syncytial virus
RWE -: Real-world evidence
VE -: Vaccine effectiveness
WHO -: World Health Organization

## 1 Introduction

Clinical endpoint randomised controlled trials (RCTs) have long been the gold standard for evaluating the safety and efficacy of medicinal products, including vaccines, and in specific situations may also be the most rapid means of generating data in support of authorisation. However, in other situations, their resource-intensive and time-consuming nature may render them either impractical, prohibitively expensive, or too slow. Moreover, RCTs may lack generalisability in some settings, including a lack of data on persons at different risk of disease. As a result, there's increasing interest in approaches that can generate timely evidence of vaccine benefit to support regulatory approval, complemented by real-world evidence (RWE) collection post-approval to confirm clinical benefit. The United States Food & Drug Administration (US FDA) and the American Medical Association defined RWE as clinical evidence on the use, benefits, and risks of medicinal products derived from real-world data, which are routinely collected data related to patient health status and healthcare delivery. RWE has been used in post-marketing vaccine safety evaluation and has an increasingly important role in assessing vaccine effectiveness, supporting initial licensure, and enabling label extensions. Here, we use a broader definition of RWE which spans a continuum of study designs. Traditional RCTs and retrospective observational studies represent the two poles of this continuum, with pragmatic trials occupying the middle ground [1]. Pragmatic trials typically use a licensed product, a randomised allocation (blinded or not), assessment of outcomes through existing databases such as electronic medical records and streamlined requirement for study conduct including safety follow-up. Pragmatic trials are highlighted as a growing and valuable approach, combining randomisation to reduce bias with real-world follow-up, broad eligibility criteria, representative populations, and routine data sources.

Regulatory pathways are available that allow approval of products without requiring a randomised clinical endpoint field trial. Protection can be inferred by evaluating immune response markers elicited by a candidate vaccine. When established correlates of protection (CoPs) exist, these can be used to support such inferences. In situations where CoPs are not available, surrogate immune markers may serve a similar role. Another approach involves the use of controlled human infection models (CHIMs), which incorporate controls and randomisation and can therefore themselves be considered a form of randomised controlled trial. CHIMS can provide significant evidence of protection. Under certain conditions, these approaches may provide sufficient reassurance of benefit before approval, reducing or, in some situations, even eliminating the need for large phase III RCTs. Indeed, there may be specific circumstances in which surrogate endpoint use is not only preferable but also the only feasible and timely option. In such cases, a pre-agreed plan for verifying effectiveness post-approval, including using RWE, if appropriate, is likely to be a requirement for licensure. During an initial meeting, we aimed to examine how RWE can be effectively leveraged to accelerate and optimise vaccine deployment across the full product lifecycle, by sharing experiences among regulators, public health authorities, and industry; identifying best practices and challenges; and defining priorities for improving data quality, transparency, collaboration, infrastructure, and capacity [2]. The current meeting's objective was to develop recommendations and initiate a framework for using diverse approaches to demonstrate vaccine benefit in the pre-licensure phase and to confirm benefit in the post-approval setting, with a focus on RWE studies and pragmatic trials.

## 2 Vaccine approval in the absence of RCT efficacy data, challenges and overview

### 2.1 Review of vaccines licensed in absence of a phase III randomised controlled efficacy trial

Danielle Craig, CEPI

The goal of more innovative registration and approval pathways is to accelerate vaccine development and access, particularly for emerging and epidemic-prone diseases where traditional phase III efficacy RCTs are not feasible. To this end, input from regulators, industry, academia, and technical expert groups should be integrated. This collaborative approach supports the Coalition of Epidemic Preparedness Initiative (CEPI)'s 100 Days Mission [3], which aims to dramatically shorten the time from outbreak detection to vaccine availability. While the 100-day target is aspirational rather than universally achievable, CEPI argues it is realistic based on precedents and requires a paradigm shift toward preparedness and proactive research conducted before outbreaks occur.

The mission framework emphasises readiness prior to outbreaks, a rapid reaction phase to develop and evaluate countermeasures, and a rollout phase in which post-authorisation use generates substantial safety and effectiveness data. This post-rollout evidence is seen as critical to supporting continued use and broader approval. CEPI highlights multiple regulatory and scientific precedents demonstrating that vaccines may, in some cases, be authorised without traditional phase III efficacy RCTs.

Where feasible, regulatory science traditionally requires evidence of efficacy derived from well-controlled RCTs. However, for some diseases, such trials may be considered impractical or ethically challenging. Low disease incidence, unpredictable outbreaks, and limited infrastructure in affected regions can all make phase III efficacy RCTs more challenging. These realities necessitate alternative regulatory approaches that still support sound benefit–risk assessments.

Accelerated approval pathways permit earlier authorisation with deferred confirmation of clinical benefit via post-marketing required studies (Table 1). The US FDA Animal Rule, enacted in 2002 after anthrax attacks, allows efficacy to be inferred from animal models when human trials are infeasible. This may not necessarily be the most rapid path; it took the applicant (Emergent BioSolutions) approximately thirteen years to accrue the required data and to interact with the US FDA as part of a rolling submission before licensure of BioThrax was granted [4]. The European Medicines Agency (EMA) offers conditional or exceptional circumstances authorisations, and the WHO has provided guidance on the use of correlates of vaccine-induced protection [5]. Across the vaccine development lifecycle, preclinical immunogenicity and challenge studies inform early decisions, followed by clinical safety and immunogenicity assessments.

**Table 1:** Accelerated approval pathways.

Pathway	RA	Description
Approval under exceptional circumstances	EMA	A type of marketing authorisation granted when it is not possible for the applicant to provide comprehensive data on a medicine's safety and efficacy under normal conditions of use.
Conditional marketing authorisation	EMA	A pathway that allows approval of a medicine before comprehensive clinical data are fully available, provided that the medicine addresses an unmet medical need, the benefit–risk balance is positive, the applicant is likely to provide the required comprehensive data after authorisation, and immediate patient access offers more benefit than the risk of incomplete data.
Animal rule	US FDA	Allows approval of drugs or biologics based on well-controlled animal studies when human trials are unethical or impossible, specifically for products aimed at preventing or reducing serious harm from lethal or permanently disabling chemical, biological, radiological, or nuclear threats.
Accelerated approval	US FDA	A regulatory mechanism to approve drugs for serious or life-threatening conditions earlier than traditional approval by relying on surrogate endpoints/measurable markers reasonably likely to predict clinical benefit.
Emergency use listing	WHO	A risk-based regulatory pathway used to assess and list unlicensed vaccines, therapeutics, and diagnostics during public health emergencies. Its purpose is to expedite availability of products based on essential data regarding quality, safety, and efficacy when a rapid response is needed.

RA – Regulatory authority; EMA – European Medicines Agency; FDA – United States Food & Drugs Agency; WHO – World Health Organization.

Scientific approaches that have been used to estimate vaccine efficacy without a clinical endpoint field trial include: use of established CoPs; clinical immunobridging based on understood but not fully validated immune-effectiveness relationships; identification of protective antibody titres using passive transfer studies with human immune sera in animal challenge models to provide protection against the challenge agent; actively vaccinated and protected animals in challenge studies; and use of CHIMs supported by seroepidemiological data. Numerous vaccines have been authorised using these approaches.

Returning to the 100 Days Mission, the focus shifts to the role of RWE. CEPI emphasises that emergency or conditional authorisation should not end clinical development. Controlled trials for additional populations remain important, but large-scale real-world data are often essential to validate predicted effectiveness, monitor safety, and inform regulatory decisions. Ultimately, the goal is to support progression from emergency use to full approval, using structured frameworks that integrate RWE alongside traditional and alternative data sources.

## 3 Alternative approaches to RCT efficacy data to demonstrate vaccine benefit for initial approval

### 3.1 Summary and insights from the EMA workshop (24-25 Nov 2025) on the use of animal models

Marco Cavaleri, EMA

EMA, together with WHO and CEPI, organised an international regulatory workshop to evaluate the appropriateness of using nonclinical animal models to support initial approval of vaccines and other medical countermeasures for future public health threats [6]. The meeting, with broad global regulatory participation, addressed whether current regulatory pathways relying on animal data are scientifically robust or require adjustment. The scope extended beyond vaccines to the full spectrum of chemical, biological, radiological, and nuclear threats, where clinical efficacy trials are often infeasible or unethical, making animal models indispensable.

Discussions were partly motivated by recent experience with an antiviral (tecovirimat) approved based on pivotal animal models data that later failed to demonstrate efficacy in randomised trials during the mpox outbreak [7,8]. This prompted critical reflection on whether shortcomings stemmed from adequacy of the models or from misinterpretation of what animal data can and cannot predict. Overall, regulators agreed that animal models remain essential and often the only viable option, but their design, execution, and interpretation require greater rigour. Key considerations include the choice of species, disease relevance, challenge strain, dose, route of administration, timing, and reproducibility across laboratories. Non-human primates were widely viewed as the most predictive models, despite ethical and logistical constraints.

The workshop highlighted different regulatory approaches, including the FDA Animal Rule and the EMA's more flexible exceptional circumstances pathway, both of which require robust human safety data and post-approval requirements or commitments to generate clinical evidence when possible. Case studies illustrated diverse bridging strategies, such as immunogenicity modelling for an Ebola prime–boost vaccine and definition of antibody thresholds for chikungunya vaccines supported by passive transfer studies and seroepidemiology. These examples underscored that immune markers may predict short-term protection but are not universally transferable across platforms, pathogens, or time horizons.

Participants emphasised an “entirety of evidence” approach combining animal data with clinical immunogenicity, epidemiology, and RWE, while acknowledging limitations and bias. RCTs including the option of pragmatic trials remain the preferred post-approval standard, but carefully designed observational studies may be necessary when trials are infeasible. Overall, the workshop concluded that animal-model–based pathways remain valid, provided they are applied cautiously, transparently, and complemented by post-approval evidence generation.

### 3.2 Developing a Group B Streptococcus vaccine for maternal immunisation: challenges for clinical development in a low-incidence, high impact infectious disease setting

Lidia Oostvogels, Minervax

Group B Streptococcus (GBS) is a prevalent human commensal that colonises approximately 20% of individuals at any given time, with most people carrying GBS at some point during their lifetime. Colonisation is usually asymptomatic, yet it poses significant risk during pregnancy, as vertical transmission can lead to severe outcomes including stillbirth and invasive GBS disease in neonates and young infants. Despite widespread screening and the routine use of intrapartum antibiotic prophylaxis (IAP) in high-income countries, current preventive strategies remain insufficient, especially for late-onset disease. Invasive GBS disease still occurs in approximately 0.5 – 2.5 per 1000 live births, highlighting persistent residual risk even in settings with robust healthcare infrastructure. Most low- and middle-income countries have little or no GBS screening or IAP.

The medical need for improved prevention of GBS disease in neonates has led to strong global support for developing maternal GBS vaccines. The scientific rationale for maternal immunisation is well established. Naturally acquired antibodies against multiple GBS antigens are common due to lifelong exposure, and low maternal antibody levels have been associated with increased risk of invasive neonatal disease. Vaccination during pregnancy aims to boost maternal immunity and enable efficient transplacental transfer of functional antibodies, providing direct protection to the pregnant person and passive protection to the infant during the first months of life [9]. Candidate vaccine antigens include capsular polysaccharides from multiple serotypes, conserved surface proteins, such as proteins from the Alpha-like family, pilus proteins, and BibA, with functional antibody activity in cord blood considered a critical measure of protection.

The World Health Organization (WHO) has defined Preferred Product Characteristics for a maternal GBS vaccine, emphasising prevention of laboratory-confirmed GBS stillbirth and invasive neonatal disease, administration during the second or third trimester, and safety comparable to other vaccines recommended in pregnancy [9]. A single-dose regimen is preferred, with target efficacy of 80% against stillbirth and invasive disease and coverage of more than 90% of circulating invasive GBS isolates [10]. Global health bodies, including WHO advisory groups, Gavi, and national immunisation committees, have articulated support for accelerated licensure pathways and post-licensure evidence generation to enable timely access.

A major challenge in GBS vaccine development is the low incidence but high impact nature of invasive disease. A conventional phase III efficacy trial powered on clinical endpoints would require at least 60,000 mother-infant pairs in high-incidence settings, hence requiring 120,000 trial participants (60,000 mothers and their 60,000 infants). Such trials are further complicated by diagnostic challenges, including antibiotic administration before blood cultures, and difficulty attributing causality in stillbirth (considering limited autopsy feasibility) or preterm birth. Consequently, alternative licensure pathways are being pursued, based on robust safety data and a serological threshold of risk reduction [SToRR]) i.e., immunological endpoints predictive of clinical benefit [11,12], followed/supported by Real-World Data (RWD) and Real-World Evidence (RWE). Several vaccines are in clinical development, including polysaccharide-based and protein-based candidates. Developers (Minervax, Pfizer, Inventprise) are engaging with regulators on this surrogate efficacy approach, with RWE expected to play a critical role post-licensure.

### 3.3 Evidence on vaccine benefit from human infection models: insights and considerations

Robert Read, University of Southampton

CHIMs, while historically controversial, are now conducted within a robust ethical framework [13], emphasising minimised risk, qualified research centres, reversibility of infection, and high-quality informed consent. CHIMs can be disease models, which deliberately induce symptomatic illness and are often more persuasive to regulators. Alternatively, CHIM may utilise colonisation or infection-only outcomes, which study acquisition and persistence of pathogens without causing clinical disease.

Within the EU-funded PERISCOPE consortium a *Bordetella pertussis* nasopharyngeal colonisation model was developed. Given ethical and practical constraints associated with inducing pertussis disease, particularly the unpredictable duration and severity of cough, the consortium selected a colonisation model [14]. Volunteers with low baseline anti–pertussis toxin IgG were intranasally inoculated with a carefully titrated dose of *B. pertussis*. A dose-ranging inpatient pilot study identified 10^5^ colony-forming units as sufficient to achieve colonisation in approximately 80 percent of participants without significant symptoms [15]. Colonisation was transient, safely cleared with azithromycin, and associated with no detectable environmental shedding or secondary transmission. Nasal wash culture proved the most sensitive diagnostic method, outperforming commonly used throat swab PCR assays.

Subsequent outpatient studies confirmed safety and reproducibility, demonstrated lower colonisation rates in unselected populations, and showed resistance to reinfection at three months, indicating induction of protective immunity [16,17]. Immunological analyses revealed associations between higher pre-challenge serum pertussis toxin-specific and filamentous hemagglutinin-specific IgG and mucosal IgA responses and reduced colonisation. After challenge seroconversion and memory B cell responses to infection were observed. Cellular correlates suggested roles for IL-22– and IL-17–producing T cells in protection [18].

The established model was then used to evaluate a live attenuated intranasal pertussis vaccine, which reduced colonisation likelihood and density [19]. Subsequently, secretory IgA has been shown to be a key correlate of the protection observed in that study (Hill A., personal communication). Overall, the work demonstrates that a safe, ethically robust human pertussis colonisation model can generate valuable insights into transmission biology, immune CoPs, and vaccine efficacy.

In light of this evidence, the Vaccines and Related Biological Products Advisory Committee (VRBPAC) of the FDA reached a consensus during its 20 September 2024 meeting that CHIMs can be used to assess the efficacy of new *Bordetella pertussis* vaccines [20].

### 3.4 Evidence on vaccine benefit based on correlates of protection: insights and considerations

Phil Krause, Independent Consultant

During outbreaks, CoPs are often treated as the only feasible path to licensure, however, this assumption is frequently incorrect. Outbreaks can provide unique opportunities to conduct efficacy RCTs, especially when vaccines are expected to be highly effective. When true efficacy is in the range of 85–95%, statistically robust efficacy estimates can be obtained with relatively few cases, allowing even phase IIb or smaller trials to contribute useful efficacy information, indeed potentially even sufficient information to support licensure. In such settings, placebo-controlled efficacy RCTs can yield definitive results faster than attempts to establish CoPs, which may require large cohorts of vaccinated people to observe sufficient breakthrough cases. Establishing CoPs is most feasible for less effective vaccines, because failures in vaccinated individuals are needed to correlate immune markers with lack of protection. For highly efficacious vaccines, CoPs may be impossible to define due to the absence of failures.

CoPs and immunobridging are qualitatively different approaches to licensure. A CoP is a marker of immune function that statistically correlates with protection against infection after vaccination [21], whereas immunobridging seeks to demonstrate that a new vaccine induces immune responses qualitatively and quantitatively similar to those of a proven vaccine. Immunobridging is easier to implement and is commonly used to extend indications to new populations, dosing regimens, formulations, or manufacturing changes, and sometimes to support approval of new vaccines. However, its validity depends on strong assumptions about shared mechanisms of protection, particularly when cellular immunity plays a major role. Antibody-based bridging is often used because antibodies are easier to measure, even when cellular immune responses may be more important for protection.

Examples include the pneumococcal conjugate vaccines, where successive products have been approved via immunobridging to earlier vaccines with demonstrated efficacy. This has raised concerns about “immunogenicity creep,” whereby non-inferiority margins can allow incremental declines in immune responses as more serotypes are added. Under the assumption that an increasing number of serotypes leads to ever decreasing immunogenicity (at least for some serotypes), this approach may eventually risk approving vaccines that are less effective than their predecessors. Potential solutions are use of totality of evidence, use of post-licensure data (including observational data) from the immediately preceding vaccine construct to verify efficacy, use of tighter non-inferiority margins (although this risks the possibility of failing to license an effective vaccine), use of earlier pneumococcal vaccines with higher efficacy as comparators, and more regulatory reliance on post-licensure effectiveness studies at the time of initial vaccine licensure, particularly when no safety concerns have been raised.

## 4 Panel discussion: dealing with uncertainty in vaccine benefit at initial approval and its consequences for post-marketing activities

Moderator: Laurence de Moerlooze

Panellists: Dean Smith, Health Canada; Hector S. Izurieta, FDA; Brenda Gomes Valente, ANVISA; Marco Cavaleri, EMA; Danielle Craig, CEPI; and Robert Read, University of Southampton.

### 4.1 Breadth versus depth of protection

For pneumococcal vaccines, an implicit question concerns the balance between expanding serotype coverage and maintaining robust immune responses to already covered serotypes. As valency increases, newly added serotypes may be less prevalent, because the serotypes most frequently causing disease are already targeted by earlier vaccines. This raises the possibility that losses in immunogenicity to previously common serotypes could outweigh the marginal benefits of adding rarer ones.

Data from the UK suggest that different pneumococcal serotypes require different antibody levels for protection, with serotype 3 requiring substantially higher titres than others [22], although this observation was not reproduced in other European countries [23]. Some serotypes may meet noninferiority criteria yet perform less well clinically and, vice-versa, others do not meet noninferiority criteria but protect well. These observations reinforce that current immunogenicity thresholds are not absolute CoPs but rather benchmarking tools with known limitations.

The central challenge, therefore, is determining when reduced immunogenicity becomes clinically meaningful. This question cannot be answered without protection-focused data, difficult to obtain for rare outcomes like invasive pneumococcal disease in infants. Furthermore, protection against clinical outcomes may have to be complemented with impact on carriage in case sufficient to maintain indirect effects on disease.

### 4.2 Postmarketing evidence as a cornerstone of risk management

The discussion repeatedly returned to the role of postmarketing studies in resolving residual uncertainty. Participants broadly agreed that vaccines approved based on immunobridging or other surrogate markers should not be viewed as having completed their evidentiary journey at licensure. Indeed, as contemplated by existing regulatory frameworks, approval marks a transition into a new phase of evidence generation.

Observational effectiveness studies were recognised as valuable, especially when supported by strong national healthcare databases, as seen during the COVID-19 pandemic. Such studies can provide rapid, population-level insights that would be impossible to obtain through RCTs alone. However, their limitations were also acknowledged, including susceptibility to confounding.

Another practical complication is that postmarketing requirements/commitments relying on observational RWE depend on vaccine uptake. Several COVID-19 vaccines approved with requirements for effectiveness studies were never widely used, making data generation impossible. This reality complicates reliance on RWE postmarketing evidence.

For this reason, there was strong support for pragmatic randomised trials where feasible. These designs can offer a balance between methodological rigour and real-world applicability. Participants emphasised that no single study design should dominate; instead, regulators should adopt a portfolio approach, selecting methods based on disease epidemiology, feasibility, timeliness, and risk.

### 4.3 Lessons from historical use of surrogate markers

The panel reflected on historical precedents where vaccines approved on surrogate markers proved highly effective. Hepatitis B was cited as a clear success, supported by immune globulin studies that established a biologically sound CoP. Meningococcal C vaccination in the UK provided another instructive example: the vaccine was approved and deployed in the context of an unmet medical need based entirely on serum bactericidal antibody data and was confirmed to be highly effective post-approval based on observational studies.

However, subsequent analysis revealed that protection at the population level was enhanced by reduced carriage and herd immunity. A broader point emphasised by several speakers was that correlates often reflect immune processes that occur at the same time, rather than a single biomarker univocally mediating protection. For example, antibody binding assays may be used where neutralisation is the mechanism of action, but humoral markers may succeed as surrogates even if cellular immunity or other mechanisms are central.

These examples support continued use of surrogate markers where scientifically justified, while underscoring the importance of post-licensure learning and humility about mechanistic assumptions.

### 4.4 Geographic generalisability of real-world evidence

A final theme concerned whether RWE generated in one country can inform regulatory decisions elsewhere. The consensus was that it often can and should. COVID-19 provided compelling examples, particularly high-quality observational studies from Scandinavian countries that influenced decisions globally. Regulators from Brazil and Canada reported that effectiveness data from other jurisdictions aligned well with their own national experience.

At the same time, participants cautioned against assuming universal generalisability. Differences in epidemiology, circulating strains, and healthcare systems do matter. The goal should be judicious extrapolation whenever appropriate rather than automatic assumption that data will be generalisable to new settings. Building global infrastructure for high-quality surveillance and timely data sharing was identified as a priority, both to improve confidence in extrapolation and to reduce unnecessary duplication.

In summary, the discussion converged on a shared regulatory philosophy: surrogate immune markers and immunobridging remain essential tools for timely vaccine approval, when clinical trials with disease endpoints are unfeasible or would take too long. However, they must be applied with an awareness of their limitations, supported by clear boundaries to prevent cumulative erosion of performance standards, and embedded within a lifecycle approach that prioritises robust postmarketing evidence.

## 5 RWE to confirm vaccine benefit

### 5.1 Real-world evidence to confirm vaccine benefit: mpox vaccine

Victoria Jenkins, Bavarian Nordic

MVA-BN (Jynneos ®, Imvamune ®/Imvanex ®, Bavarian Nordic A/S) is a third-generation non-replicating smallpox and mpox vaccine developed as an alternative to earlier replicating vaccines contraindicated in certain populations such as immunocompromised individuals. Because smallpox had been eradicated at the time of development, traditional efficacy trials were not feasible, and initial licensure in Europe, Canada, and, later, the United States, relied on extensive preclinical animal challenge studies and clinical immunogenicity and safety data. Non-human primate studies demonstrated strong protection against monkeypox challenge, with survival rates of 80–100% in vaccinated animals compared with poor survival in controls [24]. More than 9000 human individuals participated in over 20 clinical studies, generating substantial safety data, including in risk populations such as people living with HIV or atopic dermatitis [[25], [26], [27], [28], [29], [30], [31], [32], [33], [34]].

RWE became critical during the global 2022 mpox outbreak, when approximately one million doses were administered in the United States. Post-marketing surveillance confirmed a favourable safety profile consistent with clinical trials, with mostly mild injection-site reactions and rare serious adverse events [[35], [36], [37], [38], [39]]. Observational effectiveness studies from several countries showed vaccine effectiveness (VE) ranging from 35 to 89% after one dose and 66–90% after two doses [37], informing outbreak response strategies and enabling label updates. Although this observational study did not change the indication, a label update was accepted by EMA and included in the 5.1 Pharmacodynamic properties in the EU SmPC. Similarly, Switzerland, UK and Canada also updated their label.

Despite these findings, clear immune CoPs for mpox remain undefined. Vaccinia binding antibodies were used for immunobridging in vaccine approval and have been shown to be associated with protection across vaccine doses and platforms [40]**.** Cellular immune responses and broader epitope recognition likely contribute to protection [41], but are operationally more complex to obtain and more difficult to assess in a standardised way, underscoring the importance of RWE to better understand vaccine performance when conventional immunological markers are insufficient.

### 5.2 Real-world evidence to confirm vaccine benefit: meningococcal B vaccine

Ilaria Bartalesi, GSK

Invasive meningococcal serogroup B disease is rare but severe, with rapid onset, high case fatality rate, and substantial long-term sequelae, particularly affecting infants and adolescents. Due to low incidence of the disease, phase III RCTs have assessed vaccine efficacy based on CoPs. Hence, initial approval of 4CMenB (Bexsero®, GSK) in 2013 was based on safety, immunogenicity, and predicted strain coverage using the Meningococcal Antigen Typing System assay [42], with subsequent post-authorisation commitments to generate real-world effectiveness (RWE) data post-marketing.

A key example of RWE generation occurred in the UK, where the vaccine was introduced into the national infant immunisation programme (NIP) in 2015 [43]. A post-approval commitment study was carried out in the context of the UK infants NIP to generate real-world evidence on vaccine effectiveness and the protocol was discussed with EMA prior to study start. This large observational study involving approximately two million infants over a 3-year period [43] demonstrated significant vaccine impact and supported updates to the European product label, including changes to dosing schedules and inclusion of real-world vaccine impact data. Subsequently, extensive global use of 4CMenB enabled the generation of additional RWE across multiple geographies, confirming reductions in disease incidence and VE in diverse populations. A systematic literature review was performed of all real-world studies on 4CMenB vaccine effects on meningococcal serogroup B disease, five studies reporting estimates on 4CMenB vaccine effectiveness and impact were reviewed. These studies showed great diversity in population, vaccination schedule and analytic methods mainly due to diversity in vaccination strategies and recommendations in the study settings. Directed by this diversity, no quantitative pooling methods to synthesise findings could be applied; instead, studies were critically assessed based on their applied methods. Reported VE estimates ranged from 59% to 94% and VI estimates ranged from 31% to 75%, representing diverse vaccination schedules, age groups, and analytic methods [44].

Regulatory acceptance of RWE varied across geographies due to different regulatory requirements, with higher acceptance when study protocols were discussed with regulatory authorities in advance. Differences in regulatory requirements and lack of global alignment limited consistent inclusion of RWE in product labels. Overall, the experience highlights the critical role of RWE in evaluating vaccines for rare diseases or those with unpredictable epidemiology, informing immunisation policy, and supporting adaptive regulatory and public health decision-making.

### 5.3 Real-world evidence to confirm vaccine benefit: ebola vaccines

Phil Krause, Independent Consultant

The efficacy of an Ebola vaccine was derived from a randomised ring vaccination trial in Guinea during the 2014–2015 West African outbreak and a subsequent large observational study in the Democratic Republic of the Congo (DRC). After earlier trials in Liberia and Sierra Leone failed to generate informative efficacy data due to logistical constraints and declining incidence, WHO implemented an adaptive ring vaccination design in Guinea, inspired by smallpox eradication strategies. In this approach, contacts and contacts of contacts of confirmed Ebola cases were identified and randomised to immediate vaccination or vaccination delayed by 21 days. The primary analysis focused on Ebola cases occurring between days 10 and 31 post-randomisation, allowing time for vaccine-induced protection. Deliberate delays would be unethical for licensed vaccines because the vaccine would be withheld from the placebo-arm. However, in the ring trial no safe and efficacious licensed vaccine existed, rendering the trial ethically acceptable [45].

Across 98 clusters, no cases occurred among immediately vaccinated individuals after day 10, whereas ten cases occurred in four clusters in the delayed group [46]. Although the trial missed its prespecified statistical threshold, multiple analyses, including intention-to-treat and per-protocol comparisons, consistently indicated substantial vaccine protection. These findings supported global licensure of the vaccine.

Further evidence came from a large observational cohort during a prolonged outbreak in the DRC, conducted under extreme insecurity [47]. More than 250,000 individuals received ring vaccination. Despite a nonrandomised design, incidence patterns closely mirrored those observed in Guinea, with marked reduction in cases after approximately 10 days post-vaccination. The larger dataset enabled subgroup analyses, suggesting efficacy across age groups and in pregnant women. Overall, replication of randomised trial findings in a real-world, high-risk setting strengthened confidence in VE and illustrated how well-designed observational studies can credibly extend evidence from randomised trials.

### 5.4 Real-world evidence to confirm vaccine benefit: chikungunya vaccine

Victoria Jenkins, Bavarian Nordic

Chikungunya is an arthropod-borne alphavirus transmitted by Aedes mosquitoes that typically presents as an acute febrile illness with fatigue, myalgia, and severe joint pain. Clinical diagnosis is challenging because symptoms overlap with other mosquito-borne diseases such as dengue, leading to underdiagnosis and underestimation of disease burden. Although mortality is low, morbidity is substantial, with approximately 40% of infected individuals developing chronic joint pain [[48], [49], [50]]. CHIKV VLP (Vimkunya ®, Bavarian Nordic A/S), a virus-like particle vaccine adjuvanted with aluminium, was recently approved in the US, EU, and UK for individuals aged 12 years and older. Due to the sporadic and unpredictable epidemiology of chikungunya, conducting pre-licensure efficacy RCT was not considered to be feasible. Regulatory authorities therefore accepted immunogenicity as a surrogate endpoint for licensure, with post-authorisation efficacy studies required.

A key step was establishing a surrogate CoP. Passive transfer studies of serum from immunised humans into non-human primates demonstrated that neutralising antibody titres above defined thresholds were strongly associated with protection against viremia, leading regulators to accept an NT80 threshold of 100 as predictive of clinical benefit [51]. Immunogenicity RCTs showed robust and rapid immune responses, with seroprotection rates of 98% in individuals aged 12–64 years and 87% in those aged ≥65 years by day 22, alongside a favourable safety profile [52,53].

Post-authorisation commitments include pregnancy registries, paediatric RCTs for immunogenicity and safety, long-term follow-up of immune duration, safety and immunogenicity of a booster dose, and a phase IIIb efficacy RCT, designed to be triggered by outbreaks. However, operationalising such studies posed major challenges due to unpredictable outbreak locations, limited surveillance sensitivity, localised transmission, and ethical and logistical constraints. Alternative observational designs were explored but found to require large populations (>10,000) and rapid response capacities that are currently impractical. Consequently, collaboration among industry, regulators, public health authorities, and international organisations will be essential to generate real-world effectiveness data for licensing chikungunya vaccines.

### 5.5 Real-world evidence to confirm vaccine benefit: the tetravalent dengue vaccine

Suely Tuboi and Randee Kastner, Takeda

The Dengue 401 study is a nested case-control, post-authorisation effectiveness study designed to assess the association between completion of the tetravalent dengue vaccine (TAK-003, Qdenga ®, Takeda) vaccination and hospitalisation due to virologically confirmed dengue (VCD), including severe dengue, when TAK-003 is administered as part of a vaccination program in a real-world setting. This study was initiated to address gaps in the knowledge from the pivotal Dengue 301 trial, which showed overall efficacy of the vaccine but lacked sufficient data on the efficacy against dengue serotypes 3 and 4, especially among individuals who were naive to dengue prior to vaccination. The 401 study is being conducted across Southeast Asia, where serotypes 3 and 4 are more common [54], with a three-year follow-up period to capture sufficient data.

The primary objective of the study is to estimate the association between TAK-003 vaccination and hospitalisation due to virology-confirmed dengue, including severe cases. Additionally, the study will assess risk by infecting serotype and baseline serostatus, particularly focusing on naive individuals. The study cohort consists of 70,000 participants from provinces in Southeast Asia, where TAK-003 is being administered as part of a public vaccination program. Active surveillance at participating hospitals will identify cases, which will then be matched to controls based on age and neighbourhood to account for exposure risk.

Blood samples will be taken from all cohort participants at baseline and stored. Cases (hospitalised dengue patients) will be matched with up to ten controls. Vaccination status will be ascertained, and baseline sample will be tested for cases and controls. This study design enables the collection of effectiveness while also addressing vaccination being part of the public health program.

However, the study faces operational challenges, requiring close collaboration with health authorities, local research teams, and regulators. Ultimately, Dengue 401 aims to provide valuable data on the real-world effectiveness of the TAK-003 vaccine, with a particular focus on less prevalent serotypes and previous exposure to dengue in the population.

### 5.6 Real-world evidence to confirm vaccine benefit: next generation pneumococcal vaccines

Bradford Gessner, Independent Consultant

Pneumococcal disease affects all age groups and manifests as invasive pneumococcal disease (IPD), pneumonia and other lower respiratory tract infections (LRTI), and otitis media. For IPD, the pneumococcus is usually serotyped allowing for serotype specific efficacy and effectiveness estimates; however, IPD is rare, particularly in children, making effectiveness estimates difficult to obtain. In contrast, pneumonia and non-pneumonia LRTI account for most disease burden but are difficult to attribute to specific serotypes due to limitations in diagnostics, while otitis media lacks routine serotype confirmation because tympanostomy is rarely performed.

As the pneumococcal conjugate vaccine (PCV) benefits are well established and global introduction of PCVs is high, placebo-controlled trials are no longer considered ethical. Consequently, new generation PCVs are evaluated against current standard-of-care PCVs, making traditional efficacy trials impractical logistically and financially. Instead, regulatory agencies rely on immunogenicity-based noninferiority pathways, comparing serotype-specific IgG responses of new vaccines to earlier generations. This approach has enabled licensure of higher-valency PCVs but has important limitations. Immunogenicity endpoints (based on IgG geometric mean concentrations and percent of vaccinated individuals above a predefined threshold of 0.35 μg/ml in children and OPA values in adults) are accepted largely because they can be bridged to historic RCTs demonstrating clinical efficacy. Yet their acceptance has limitations. For example, in paediatrics the predefined IgG threshold of 0.35 μg/ml a) is not an established CoP; b) was determined only for IPD; c) was determined only for the initial seven serotypes included in PCV7; d) focuses narrowly on circulating IgG, ignoring mucosal, cellular, and innate immunity; e) does not apply to carriage and transmission; f) is based on an aggregate value for all serotypes combined and across three different populations in which the original PCV7 efficacy studies were conducted; and g) is based on a specific 2-4-6-12 month infant immunisation schedule.

Even if clinical vaccine efficacy studies were feasible (practically, financially, ethically), results can vary with local serotype distribution. By contrast, immunogenicity provides serotype-specific information independent of epidemiology. However, regulatory interpretation of immunogenicity data and the “totality of evidence” can be inconsistent, leading to divergent licensure decisions, schedule restrictions, and extensive post-authorisation study requirements that may be difficult to fulfil in a fragmented and rapidly evolving PCV market. Additionally, narrow regulatory endpoints may substantially underestimate public health impact. For example, data from adult trials show that reductions in all-cause LRTI far exceed those observed for etiologically confirmed vaccine-type IPD or etiologically confirmed pneumococcal pneumonia alone [[55], [56], [57]].

Current licensure frameworks based on immunogenicity comparisons, while practical, may lead to efficacious PCVs failing to meet licensure criteria. Potential improvements include redefining immunogenicity endpoints, recognising that noninferiority failures for infant doses are less important than those for the toddler dose for public health impact, accepting broader outcomes such as carriage or all-cause disease, identifying CoPs, and integrating pragmatic or observational post-licensure studies to better align regulatory decisions with real-world public health benefit.

### 5.7 Real-world evidence to confirm vaccine benefit: COVID-19 vaccine effectiveness through the pandemic and beyond

Alexander Allen, UKHSA

The COVID-19 pandemic provided a unique case study due to rapid vaccine development including demonstration of high efficacy of candidate vaccine in RCTs, the emergence of novel platforms such as mRNA, and the highly dynamic epidemiology of SARS-CoV-2 with frequent variant turnover. These factors often made reliance on RCT impractical for next generation vaccines to inform timely vaccination policy, necessitating robust observational approaches. The UK COVID-19 vaccination programme evolved from mass rollout of AstraZeneca and Pfizer/BioNTech vaccines in 2020–2021 to increasingly targeted booster campaigns focused on older adults, care home residents, and immunosuppressed individuals. Vaccine policy is informed by the UK Health Security Agency evidence provided to the Joint Committee on Vaccination and Immunisation, which advises government ministers.

Key post-licensure evaluation methods include test-negative case-control studies, which were central for estimating VE against hospitalisation, helping to reduce bias related to healthcare-seeking behaviour [[58], [59], [60]]. These analyses showed consistent peak effectiveness of booster doses of around 40–50% against severe disease across variants and campaigns, with more sustained protection observed in recent booster rounds, potentially reflecting viral stabilisation or improved vaccine matching. However, low disease incidence reduced statistical power during some periods.

Age-discontinuity analyses were used as quasi-experimental designs to assess mortality impacts around age-based eligibility thresholds. These suggested modest reductions in COVID-19 mortality, likely influenced by outcome misclassification and healthy vaccinee bias, though stronger effects were demonstrated for other vaccines such as RSV. Vaccine safety surveillance relied on spontaneous reporting systems complemented by self-controlled case series analyses using linked national data, enabling robust assessment of rare adverse events. Overall, RWE played a critical role in adapting vaccination strategies while continually refining methods to address bias and confounding.

### 5.8 Real-world evidence to confirm vaccine benefit: updating COVID-19 vaccines

Kyla Hayford, Pfizer

Phase IV real-world VE studies played an important role in seasonal COVID-19 vaccine strain selection, alongside preclinical and clinical data. COVID-19 has transitioned from pandemic to endemic status yet continues to cause substantial morbidity and mortality comparable to influenza, even with relatively low vaccine uptake. This reduced incidence, however, poses challenges for timely and adequately powered VE assessments.

A continuous, annual framework for vaccine updating is necessary that integrates variant surveillance, preclinical immunogenicity studies, clinical immunobridging, and post-licensure VE evaluations. Since initial authorisation, multiple variant-adapted formulations have been introduced to address immune escape driven by viral evolution. Real-world VE against COVID-19 hospitalisation has remained generally sustained across updates, though with notable variability influenced by confounding, background population immunity, waning, and antigenic drift [[61], [62], [63], [64], [65]].

Using the transition from XBB.1.5 to JN.1 as an example, concordance has been shown between preclinical reductions in neutralisation, diminished clinical immune responses, and decreased real-world VE, which prompted selection of a JN.1-adapted vaccine. Subsequent data demonstrated improved immunogenicity and higher VE against hospitalisation [63]. Similar benefits were observed with the KP.2 vaccine in multiple test-negative design studies [65], though VE estimates varied by population characteristics, timing, and circulating variants, highlighting the importance of understanding heterogeneity rather than focusing solely on point estimates.

Integrating preclinical, clinical, and RWE has enabled timely adaptation of COVID-19 vaccines to antigenically divergent variants. Real-world VE studies both initiate and validate the strain selection cycle, remaining critical despite study limitations, declining case numbers and surveillance capacity, and underpin informed regulatory and public health decisions for ongoing seasonal vaccination strategies.

## 6 Pragmatic randomised controlled trials for vaccine effectiveness

While RCTs remain the gold standard for efficacy assessment, their use becomes challenging after licensure, particularly if placebo controls are no longer ethical or feasible. Historically, observational studies have been the main approach for post-licensure vaccine evaluation. Observational studies can address questions that pre-licensure RCTs cannot, especially regarding rare safety events, outcomes such as hospitalisation and death that require very large populations, or outcomes in specific risk groups that are not large enough to enrol in pre-licensure RCTs, and indirect effects. However, such studies are vulnerable to confounding, bias, and uncontrolled type I error, particularly when analyses are not prospectively defined. Bias associated with typical observational studies can be reduced, though not eliminated, through prospective protocols, use of designs that address immortal time bias, measured and unmeasured confounding, including through better matching, internal controls, falsification outcomes, and replication across studies. Test-negative designs may help control for health-seeking behaviour, although their limitations were evident during the COVID-19 pandemic.

To improve causal inference while preserving feasibility and fairness, pragmatic randomised trials can provide the advantages associated with very large studies, while also addressing some of these deficiencies of observational studies. Examples include randomising vaccination appointment timing within defined risk groups, cluster randomisation by location, or randomising immediate versus delayed vaccination, as used in Ebola studies (as part of the rollout system and not introduced in order to have a study comparator). Such approaches could generate robust safety and efficacy data rapidly while ensuring equitable access during limited supply.

Pragmatic trials could leverage existing healthcare and electronic record systems, creating a hybrid between randomised and observational studies without requiring full clinical trial infrastructure, thus, facilitating use of larger, more generalisable trials with improved timeliness. Ethical considerations include limited supply, delayed access rather than denial of vaccination, and simplified informed consent. A number of examples of the use of pragmatic trials followed, outlining both their strengths and limitations.

### 6.1 Lessons from a pragmatic randomised trial: high-dose vs. standard-dose influenza vaccine against severe clinical outcomes (FLUNITY-HD)

Joshua Nealon, Sanofi

The high-dose influenza vaccine, containing four times the amount of antigen of standard formulations, had previously demonstrated superior immunogenicity and relative efficacy against laboratory-confirmed influenza-like illness in a classical post-licensure RCT when compared to a standard-dose vaccine [66,67]. However, that trial – as is typical for classical randomised trials - relied for feasibility reasons on mild clinical disease as a primary endpoint, limiting its perceived clinical relevance. Substantial observational evidence and biological plausibility suggested influenza vaccination could reduce severe outcomes such as hospitalisations and cardiovascular events [[68], [69], [70], [71], [72], [73]], but such data are insufficiently robust, due to bias and lack of reproducibility, to meaningfully affect vaccination policy.

To address these limitations, investigators implemented a pragmatic randomised trial embedded in routine care, leveraging national health registries [[74], [75], [76], [77]]. Older adults were individually randomised at vaccination sites to receive either high-dose or standard-dose vaccine, with minimal inclusion and exclusion criteria to maximise generalisability. Follow-up relied entirely on routinely collected healthcare data, creating a hybrid design that preserved randomisation while using real-world outcome ascertainment. After an initial feasibility phase in Denmark, the study expanded to include Spain (Galicia) to achieve adequate power, resulting in a combined population of nearly half a million participants across multiple influenza seasons.

The primary endpoint was hospitalisation for influenza or pneumonia, with secondary endpoints including cardiorespiratory hospitalisations, laboratory-confirmed influenza, all-cause hospitalisation, and mortality, tested hierarchically. The study demonstrated modest but statistically significant relative reductions in several severe outcomes [78], consistent with expectations for incremental benefits over an already effective standard vaccine. Limitations included lack of blinding, heterogeneity across sites, and reliance on diagnostic coding. Overall, the trial illustrated that large-scale pragmatic randomisation is feasible, offers superior causal inference compared with observational studies, and if RCT data are required may be essential as an extension for confirming small but clinically meaningful benefits of vaccines in real-world settings.

### 6.2 Lessons from a pragmatic randomised trial: RSV vaccine effectiveness against hospitalisations

Bradford Gessner, Independent Consultant

The Dan-RSV study is an ongoing large-scale, pragmatic, individually randomised trial evaluating the real-world effectiveness of an RSV prefusion F vaccine in adults aged 60 years and older in Denmark. The study was motivated by limitations of the phase III licensure trial [[79], [80], [81]], which demonstrated high efficacy against mild RSV disease but was based on a small number of events and lacked data on severe outcomes, all-cause endpoints, and key subgroups. Although multiple observational studies suggested high effectiveness against hospitalisation, these findings required confirmation through randomised evidence [[82], [83], [84], [85]].

The Dan RSV study used nationwide health registries to identify eligible individuals and sent electronic invitations to approximately 1.5 million citizens [86,87]. Participants who consented were randomised in an open-label manner to vaccination or control and followed through routine healthcare data, without additional study visits. The primary endpoint was hospitalisation for RSV-related respiratory tract disease, with alpha-controlled secondary endpoints including RSV-related lower respiratory tract disease, RSV-related and all-cause cardiorespiratory hospitalisations, and additional exploratory outcomes.

Results showed high VE for the primary endpoint, with approximately 84% reduction in RSV-related respiratory hospitalisations, closely aligning with prior observational estimates and thereby anchoring their credibility. VE was also observed across key secondary endpoints and most subgroups. Notably, while effectiveness estimates were less for all-cause than RSV specific endpoints, rate reductions were higher, indicating that RSV contributes to a larger burden of disease than is captured by laboratory testing. Exploratory analyses identified reductions in cardiovascular disease outcomes, although these findings require confirmation.

The study demonstrated the feasibility, speed, and efficiency of large-scale pragmatic randomised trials. The study was not a requirement for licensure, but because of its randomised nature could be used for regulatory updates. Regardless of regulatory considerations, the study highlights the value of pragmatic randomised trials in addressing post-licensure evidence gaps – including extending the full public health value of vaccines – while maintaining strong causal inference and high generalisability [[86], [87], [88], [89]].

### 6.3 Lessons from a pragmatic randomised trial: RSV correlates of protection in a South African vaccine effectiveness trial

Alane Izu, Wits VIDA

A phase IIIb randomised controlled trial evaluating the safety and efficacy of maternal RSV vaccination RSVpreF, in sub-Saharan Africa is ongoing, conducted during the critical post-licensure but pre-recommendation window. RSV represents a major global health burden, causing an estimated 33 million lower respiratory tract infections annually in children under five, with approximately 100,000 deaths, predominantly in low- and middle-income countries, concentrated in the first six months of life [90]. While maternal RSV vaccination demonstrated high efficacy in phase III trials and real-world studies from high-income settings [91,92], important evidence gaps remain for lower-income settings, particularly regarding safety and the potential risk of preterm birth.

The trial, conducted in Ghana, The Gambia, Kenya, and South Africa, is to address two key concerns: limited representation of lower-income countries in prior trials and insufficient evidence to establish or exclude a causal association between vaccination with RSVpreF and preterm births. The study design balances safety and generalisability by restricting enrolment to women with accurate gestational age assessment via first- or second-trimester ultrasound, excluding those with established risk factors for preterm birth, while otherwise maintaining broad inclusion. Vaccination can occur anytime during pregnancy according to country recommendation (24-36 weeks in all countries except South Africa, where it is 28-36 weeks). The primary objectives are to demonstrate non-inferiority for preterm birth risk (i.e., to rule out an increase in preterm births), with a predefined margin of 20%, and to assess vaccine efficacy against severe RSV lower respiratory tract infection in infants through six months of age.

The study is event-driven, powered to determine non-inferiority for risk of preterm birth (although the sample size is adequately powered for the efficacy objective as well), and plans to enrol approximately 13,000 participants. A planning study of 1000 women confirmed feasibility and refined estimates of preterm birth rates. An embedded immunogenicity subcohort will evaluate maternal and infant neutralising antibodies to explore CoPs, including in key subgroups such as women living with HIV. Robust safety oversight is provided by an independent data safety monitoring board with predefined stopping rules. Overall, the trial leverages a narrow ethical and regulatory window to generate critical safety, efficacy, and immunologic data in populations bearing the highest RSV burden. The study did not involve WHO prequalification engagement. The study was embarked upon based on a recommendation from WHO SAGE, which initially expressed concern about limited representation in the phase III pivotal efficacy trial from LMIC, and requested more data on vaccine efficacy/effectiveness from LMIC. The WHO established a working group to recommend on study design which would be best equipped to address safety and efficacy, and consensus was for a RCT. Pfizer agreed to donate the vaccines, on condition that the study could only be done in countries where the vaccine had been licensed. Pfizer subsequently assisted in submitting for licensure of the vaccine in those countries identified to be suitable for inclusion in the study. The study needed to be approved by the relevant research ethics committees and other regulatory authorities, even post-licensure of the vaccine.

### 6.4 Lessons from a pragmatic randomised trial: the power of vaccine probe analysis - the experience from Finland

Arto Palmu, FVR

Finland has a long history of pragmatic, register-based vaccine trials, supported by comprehensive national health registries and dedicated vaccine research infrastructure. Phase IV pragmatic trials have a public health rationale, aiming to estimate the total disease burden preventable by vaccination, including both recognised and unrecognised disease attributable to a target pathogen.

Using a nationwide cluster-randomised, double-blind trial of a 10-valent pneumococcal conjugate vaccine in approximately 47,000 infants, vaccine probe methodology can reveal substantial hidden disease burden [93]. While VE against IPD was high and expected, analyses of hospital discharge diagnoses identified a much larger burden of clinically suspected but culture-negative invasive disease. These syndromes, frequently coded as unspecified sepsis, were several-fold more common than IPD and were substantially reduced by vaccination, demonstrating that reliance on blood culture-confirmed endpoints markedly underestimates vaccine-preventable disease due to poor diagnostic sensitivity.

Additional analyses showed meaningful reductions in pneumonia hospitalisations, tympanostomy tube placements, and antimicrobial use to document the full vaccine-preventable diseases burden [94]. Although relative reductions for some outcomes were modest, high baseline incidence resulted in large absolute reductions, highlighting their public health importance. Despite confirmation of these results in subsequent observational studies, uptake into regulatory, policy, and scientific frameworks has been slow, potentially reflecting conservatism around established case definitions. While PCV10 licensure was initially based on immunogenicity and safety data, subsequent clinical trial data contributed to the expansion of the label. Pragmatic randomised trials and broader syndrome-based endpoints may be essential for accurately assessing full vaccine impact, particularly in settings with robust health data systems.

## 7 Barriers to and requirements for the use of pragmatic trials

Moderator: Phil Krause

Panellists: Dean Smith, Health Canada; Hector S. Izurieta, FDA; Brenda Gomes Valente, ANVISA; Marco Cavaleri, EMA; and Robert Read, University of Southampton.

The discussion opened with a shared concern: despite their promise, pragmatic trials remain underused. The panel converged on the view that this is not primarily due to a lack of interest, but rather to perceived and real structural obstacles. Although pragmatic trials involve already approved products, and EU CTR recognises the low-intervention trial, which can be used for approved products, regulators and ethics committees often apply the same expectations used for pre-licensure, investigational trials, such as blinding, extensive safety monitoring and exhaustive data collection. This mismatch creates unnecessary burden, cost, and complexity.

Risk-proportionality is central; pragmatic trials are designed to answer focused, post-approval questions at scale, and therefore require lean protocols and limited data collection, while ensuring ethically acceptable designs. When regulators demand full phase II/III-style datasets, the feasibility advantage of pragmatic trials disappears. Encouragingly, international efforts are underway to clarify regulatory expectations and promote proportional review frameworks tailored to pragmatic designs. In fact, GCP (R3) has been introduced already and requires risk-proportionality, fit-for-purpose and avoidance of unnecessary complexity, potentially providing sufficient clarification [95].

A perceived obstacle was the quality and completeness of data used in pragmatic trials. From a methodological perspective, missing data and variable data quality are unavoidable challenges, especially when trials rely on routinely collected health data not originally designed for research. These issues are less likely to be problematic for prospectively designed pragmatic trials than for typical observational studies, but it is important nonetheless to account for the potential impact of missing data. However, some limitations may have lower impact in phase 3 clinical trials compared to pragmatic trials, like poor outcome sensitivity and poor generalisability.

Countries with integrated, single-payer health systems, such as Denmark, Finland, and Norway are particularly well positioned to conduct these trials. However, this does not mean pragmatic trials are infeasible elsewhere. Randomisation itself mitigates many confounding issues that plague observational studies, and large health systems in more fragmented environments (for example, U.S. integrated delivery networks or veterans’ health systems) can still support credible pragmatic trials. The key distinction repeatedly highlighted was that randomisation, even in imperfect data environments, generally yields evidence that is perceived as more trustworthy than purely observational approaches. However, target trial emulation, a methodological framework in causal inference where researchers design and analyse observational data as if they were conducting a hypothetical RCT, is rapidly evolving [96,97], yet the lack of randomisation remains a major caveat.

Another major point of debate was the frequent reliance on open-label designs in pragmatic trials, yet blinded pragmatic trials have also proven feasible. Open-label approaches are often faster, cheaper, and more realistic in real-world settings, but they raise concerns about bias. Nevertheless, this is not a fatal flaw, but a design challenge, and it is important to note that a randomised open-label pragmatic trial is expected to provide much more reliable conclusions than a similarly designed non-randomised open-label observational study. Regulators may accept open-label pragmatic trials if potential biases are explicitly anticipated and addressed in the protocol. This could include prespecified sensitivity analyses, use of negative controls, careful endpoint selection, and transparency about limitations. The acceptability of open-label designs is closely tied to the nature of the endpoint; some outcomes (e.g., more severe disease and death) are inherently less vulnerable to bias than others and are thus preferred for both randomised pragmatic trials and traditional observational studies.

Ethical considerations formed one of the most nuanced parts of the discussion. It is essential to distinguish between situations where no proven intervention exists and those where an effective vaccine or treatment is already available. However, in settings in which a vaccine is already licensed, randomisation to no vaccine can be justified and considered ethical if vaccine is not available or accessible in the trial setting due to cost, infrastructure or supply constraints and the trial is intended to address these issues. Pragmatic trials can ethically leverage these realities, provided they are designed in collaboration with public health authorities and supported by robust risk–benefit analysis, fairness in participant selection, meaningful community engagement, and sound informed consent processes.

Poorly designed trials and poor communication about the aim of the study risk undermining trust in public health institutions and vaccines themselves. Additionally, allocation of resources to health care and initiation of interventions based on poor evidence can also be an important ethical issue. Conversely, generating credible new data to clarify uncertainty, support continued use, or identify limits of effectiveness is itself an ethical imperative.

There is a clear difference between pre-licensure and post-licensure use of pragmatic trials. Pragmatic trials may be considered unsuitable as primary evidence for initial licensure, because they are commonly long in duration, may not fulfil all outcome requirements related to specificity and there needs to be adequate confidence in safety. However, their value post-approval is substantial, especially to complement prelicensure trials which are failing to produce the evidence for introduction. Pragmatic trials are seen as particularly powerful tools with potential value for confirming accelerated approvals, animal rule authorisations, emergency or conditional approvals, and for updating product labels. They can address unanswered questions about effectiveness in subpopulations, long-term safety, or real-world performance. The challenge lies in ensuring that robust findings from pragmatic trials can be credibly incorporated into regulatory documents such as labels or summaries of product characteristics, even if they do not fit traditional evidentiary templates.

Many regulatory authorities, particularly in low- and middle-income countries, are still building experience with pragmatic and observational studies. Knowledge sharing about the entire nontraditional evidentiary journey among regulators, and between regulators and investigators is therefore critical. Sponsors and investigators can engage regulators proactively and challenge decisions on scientific grounds when warranted.

In summary, increased use of pragmatic trials will improve the evidence base for vaccine and therapeutic evaluation and thus have the potential to accelerate regulatory and recommendation decision-making for vaccines without RCT phase III efficacy data, and there is broad openness among regulators, scientists, and ethicists to expanding their use. The path forward depends less on changing fundamental principles and more on aligning expectations: proportional regulation, focused study questions, transparent handling of bias, and ethical designs grounded in real-world constraints.

## 8 Towards a framework for alternative approaches to phase III vaccine efficacy trials

### 8.1 What scenarios could support alternative approaches for vaccine licensure?

Participants broadly agreed that alternative licensure pathways are appropriate when RCTs are infeasible or unethical. It was emphasised that these circumstances are not hypothetical: regulatory systems already contain mechanisms such as accelerated approval, conditional approval, animal rule, and emergency use authorisations (Table 1). These pathways allow approval based on nontraditional evidence packages, including animal efficacy data, immunogenicity, or surrogate endpoints. Importantly, however, safety always comes first; pre-licensure safety expectations were repeatedly described as consistent across all pathways.

Accelerated and conditional approvals explicitly rely on the expectation that benefit will be confirmed after authorisation, but the nature of that confirmation is generally determined on a case-by-case basis, which allows flexibility for new approaches. Regulators pointed out that post-licensure studies are still expected to meet high standards of rigour, which makes it more difficult for observational studies to be always accepted in this setting. Observational studies, while attractive for feasibility reasons, raise the greatest concerns about bias, outcome specificity, and confounding. Many, though not all, of these concerns can be addressed using randomised pragmatic trials. Regulators therefore expect mitigation strategies, such as negative controls, and robust analytical methods, to be prespecified and justified.

A major scientific gap identified was the absence of validated CoPs for many pathogens. When established correlates exist, traditional approval is often possible, and post-licensure studies become supportive rather than essential. Advancing CoPs, especially across pathogen families, should be a strategic research priority. Without this foundational work, ambitious initiatives such as the CEPI 100 Days Mission or the WHO pathogen family approach are less likely to be successful.

Reliance on surrogate markers not established as CoPs can sometimes increase the level of uncertainty and necessitate confirmatory evidence. Confidence in surrogate-based approvals depends not only on biological plausibility but also on the quality of underlying tools, particularly assays. Validated, standardised, and well-characterised immunological assays are critical enablers of alternative pathways yet often treated as an afterthought in development planning.

Another important concern was whether evidence generated in one setting can be generalised globally. Participants questioned how post-licensure effectiveness data from high-income countries should be interpreted for low- and middle-income countries with different epidemiology, health systems, and population characteristics. Collaborative regulatory approaches were identified as essential to closing this gap.

Lack of alignment among regulators poses a major barrier, particularly regarding data expectations for alternative pathways and confirmatory studies. Sponsors described the operational burden of responding to divergent requests, which often undermines the feasibility of streamlined or lean study designs. Regulators acknowledged this problem but pointed to progress made during the COVID-19 pandemic, when unprecedented convergence around trial designs and data requirements was achieved. However, they also cautioned that such intensity is not sustainable outside emergencies.

Sponsors can take a more proactive role in driving convergence by convening joint discussions among agencies, proposing harmonised protocols, and explicitly requesting coordinated or even joint reviews. While formal global mechanisms are limited, regional regulatory forums and existing international platforms were cited as underutilised opportunities.

#### 8.1.1 Scenario 1: pre-licensure evidence in an outbreak setting

When an outbreak is sporadic, unpredictable, and time-limited, how can sufficiently rigorous evidence be generated to support vaccine use and potentially future licensure? The consensus was that no outbreak response can start from “a vial of an unknown substance.” Some minimum evidence must already exist:

− Basic safety data, often discussed as being on the order of a few thousand participants, though the acceptable size depends on disease severity.
− A presumption of potential benefit, derived from human immunogenicity, animal models, a recognised surrogate marker, or a plausible class effect.

Several study designs could realistically operate in outbreak conditions, if and when classic RCTs cannot be performed:

− Individually randomised placebo-controlled trials, including deferred-vaccination designs, were repeatedly described as ethically acceptable when equipoise exists and no proven alternative is available.
− Pragmatic or large simple trials, prioritising speed, feasibility, and collection of data relevant to rare outcomes over perfect control, while still reducing bias relative to other options, especially when resources are limited. However, this approach can only be considered when there is already strong evidence to support broad deployment of vaccine.
− Cluster-randomised trials, including ring vaccination and step-wedge designs, particularly when individual randomisation is impractical.
− Randomisation driven by scarcity, where limited vaccine supply itself justifies random allocation.

The choice among these designs was seen as contingent on outbreak size, transmission dynamics, urgency, predictability, and feasibility. Participants stressed that there is no “one-size-fits-all” approach. The Ebola experience was repeatedly cited as an example where confusion about ethics may have delayed evidence generation.

#### 8.1.2 Scenario 2: evidence generation after emergency authorisation

Once a vaccine has been authorised for emergency use, the challenge shifts to how to generate additional data to support continued use, refinement of recommendations, or full licensure.

If preclinical and early clinical data already exist, and the priority is speed, feasibility, and learning during deployment rather than de novo development, the following approaches would be feasible:

− Randomised approaches embedded in rollout, including pragmatic trials, ring vaccination or cluster allocation, when possible and ethically appropriate.
− Observational designs, including test-negative studies and cohort analyses, especially when widespread vaccination precludes randomisation.
− Banking biological samples during outbreaks to support later analyses of CoPs.

Emergency frameworks generally allow greater flexibility in the robustness of data required to support authorisation, contrasted to the evidentiary requirements for alternative approval pathways discussed in Scenario 3.

#### 8.1.3 Scenario 3: post-licensure requirements/commitments after alternative approval pathways

For vaccines approved based on immune markers or animal data, attention shifts to the purpose and design of postmarketing requirements or commitments.

Processes could be accelerated through clear regulatory guidance on the scientific goals of post-approval studies, greater flexibility for developers to propose scientifically meaningful alternatives, and use of impact studies, before-after comparisons, and ecological analyses, particularly in low-incidence settings where classical effectiveness studies are impractical.

Across all scenarios, several conclusions emerged. Evidence generation in outbreaks must be prepared in advance, with protocols, assumptions, and regulatory dialogue established before crises occur. Randomisation remains the gold standard and should be adapted creatively to real-world constraints; where randomisation is infeasible or unethical, observational studies should be considered. Safety assessment should be treated separately from efficacy, with basic safety expectations maintained regardless of study design. Surrogates and biomarkers are not second-best evidence, but essential tools when clinical endpoints are unattainable. Finally, regulatory decision-making ultimately rests with national authorities, whose primary responsibility is to their populations, while global equity and ethical consistency remain critical considerations.

Taken together, the discussion emphasised commitment to speed up access to safe and effective products developed taking advantage of evolving science to meet health needs. The goal is not perfect evidence, but timely, credible, and ethically defensible evidence that supports action in the face of urgent public health threats.

## 9 Conclusion

This meeting underscored general agreement that generation of robust efficacy data using traditional phase III RCTs is not always feasible, particularly for low-incidence diseases, outbreak settings, unpredictable disease occurrence and urgent public health needs. Participants converged on the need for flexible, science-based regulatory pathways that integrate multiple evidence sources, including RWE. Pragmatic randomised trials emerged as a promising bridge between licensure and policy recommendation, provided they are robustly designed and ethically conducted. Alignment across regulators, clearer guidance on evidentiary standards, and continued dialogue among regulators, public health institutions, and industry will be essential to operationalise these approaches and accelerate equitable vaccine access.

## CRediT authorship contribution statement

**Laurence de Moerlooze:** Conceptualization, Writing – original draft, Writing – review & editing. **Alexander Allen:** Writing – original draft, Writing – review & editing. **Ilaria Bartalesi:** Writing – original draft, Writing – review & editing. **Marco Cavaleri:** Writing – original draft, Writing – review & editing. **Miles P. Davenport:** Writing – original draft, Writing – review & editing. **Bradford D. Gessner:** Conceptualization, Writing – original draft, Writing – review & editing. **Brenda Gomes:** Writing – original draft, Writing – review & editing. **Kyla Hayford:** Writing – original draft, Writing – review & editing. **Alane Izu:** Writing – original draft, Writing – review & editing. **Hector S. Izurieta:** Writing – original draft, Writing – review & editing. **Victoria A. Jenkins:** Writing – original draft, Writing – review & editing. **Randee Kastner:** Writing – original draft, Writing – review & editing. **Phil Krause:** Writing – original draft, Writing – review & editing. **Joshua Nealon:** Writing – original draft, Writing – review & editing. **Lidia Oostvogels:** Writing – original draft, Writing – review & editing. **Arto A. Palmu:** Writing – original draft, Writing – review & editing. **Robert C. Read:** Writing – original draft, Writing – review & editing. **Carla Saenz:** Writing – original draft, Writing – review & editing. **Dean K. Smith:** Writing – original draft, Writing – review & editing. **Suely Tuboi:** Writing – original draft, Writing – review & editing. **Frank Vandendriessche:** Conceptualization, Writing – original draft, Writing – review & editing. **Marc Baay:** Writing – original draft, Writing – review & editing. **Danielle Craig:** Conceptualization, Writing – original draft, Writing – review & editing. **Pieter Neels:** Conceptualization, Writing – original draft, Writing – review & editing. **Kaatje Bollaert:** Conceptualization, Writing – original draft, Writing – review & editing.

## Disclaimer

This meeting report reflects the authors’ view and does not necessarily reflect the views or policies of their organisations.

## Declaration of Generative AI and AI-assisted technologies in the writing process

During the preparation of this work the scientific writer used the tool Fireflies to support in notetaking during the meeting. The authors reviewed and edited the content as needed and take full responsibility for the content of the publication.

## Funding

IABS received funding from CEPI, P95 and Takeda for organisation of the meeting and reporting of the outcomes.

## Declaration of competing interest

DC is a staff member of CEPI, which provided sponsorship funding for the meeting. KB, LM, and MB are employees of P95, which provided sponsorship funding for the meeting. IP is an employee of GSK. JN is an employee of Sanofi. LO is an employee of Minervax. RK and ST are employees of Takeda. VAJ is an employee of Bavarian Nordic S/A. RCR received grants from the IMI 2 Joint Undertaking, the UK National Institute for Health and Social Care Research, and ILIAD Biotechnologies. BDG received consulting fees from Pfizer, GSK, Minervax, and Sinovac. AA, AAP, AI, BGV, CS, DKS, FV, HI, IB, MC, MDP, PK, and PN declared no conflicts of interest.

## References

[1] Concato J., Stein P., Dal Pan G.J., Ball R., Corrigan-Curay J. (2020). Randomized, observational, interventional, and real-world-what's in a name?. Pharmacoepidemiol Drug Saf.

[2] Bollaerts K., Wyndham-Thomas C., Miller E., Izurieta H.S., Black S., Andrews N. (2024). The role of real-world evidence for regulatory and public health decision-making for accelerated Vaccine Deployment- a meeting report. Biologicals.

[3] Saville M., Cramer J.P., Downham M., Hacker A., Lurie N., Van der Veken L. (2022). Delivering pandemic vaccines in 100 days — what will it take?. N Engl J Med.

[4] Beasley D.W.C., Brasel T.L., Comer J.E. (2016). First vaccine approval under the FDA Animal rule. NPJ Vaccines.

[5] Immunization VaBI (2013).

[6] European Medicines Agency (2025).

[7] Ali R., Alonga J., Biampata J.L., Kombozi Basika M., Maljkovic Berry I., Bisento N. (2025). Tecovirimat for clade I MPXV infection in the Democratic Republic of Congo. N Engl J Med.

[8] National Institute of Health (NIH) (2025). NIH.

[9] Absalon J., Simon R., Radley D., Giardina P.C., Koury K., Jansen K.U. (2022). Advances towards licensure of a maternal vaccine for the prevention of invasive group B streptococcus disease in infants: a discussion of different approaches. Hum Vaccines Immunother.

[10] World Health Organization (WHO) (2017).

[11] Le Doare K., Benassi V., Cavaleri M., Enwere G., Giersing B., Goldblatt D. (2025). Clinical and regulatory development strategies for GBS vaccines intended for maternal immunisation in low- and middle-income countries. Vaccine.

[12] Vekemans J., Crofts J., Baker C.J., Goldblatt D., Heath P.T., Madhi S.A. (2019). The role of immune correlates of protection on the pathway to licensure, policy decision and use of group B Streptococcus vaccines for maternal immunization: considerations from World Health Organization consultations. Vaccine.

[13] World Health Organization (WHO) (2025).

[14] de Graaf H., Gbesemete D., Gorringe A.R., Diavatopoulos D.A., Kester K.E., Faust S.N. (2017). Investigating Bordetella pertussis colonisation and immunity: protocol for an inpatient controlled human infection model. BMJ Open.

[15] de Graaf H., Ibrahim M., Hill A.R., Gbesemete D., Vaughan A.T., Gorringe A. (2020). Controlled human infection with Bordetella pertussis induces asymptomatic, immunizing colonization. Clin Infect Dis.

[16] Diks A.M., de Graaf H., Teodosio C., Groenland R.J., de Mooij B., Ibrahim M. (2023). Distinct early cellular kinetics in participants protected against colonization upon Bordetella pertussis challenge. J Clin Investig.

[17] de Graaf H., Gbesemete D., Read R.C. (2024). Controlled human infection with Bordetella pertussis. Curr Top Microbiol Immunol.

[18] de Graaf H., Gbesemete D.F., Hill A.R., Fröberg J., Ibrahim M.M., Dale A.P. (2026). Safety, colonisation kinetics, transmissibility, and immune correlates of protection in healthy adults inoculated with Bordetella pertussis in England: a single-centre, open-label, phase 1, controlled human infection study. Lancet Microbe.

[19] Gbesemete D., Ramasamy M.N., Ibrahim M., Hill A.R., Raud L., Ferreira D.M. (2025). Efficacy, immunogenicity, and safety of the live attenuated nasal pertussis vaccine, BPZE1, in the UK: a randomised, placebo-controlled, phase 2b trial using a controlled human infection model with virulent Bordetella pertussis. Lancet Microbe.

[20] U.S. Food & Drug Administration (FDA) (2024). Vaccines and related biological products advisory committee meeting.

[21] Plotkin S.A., Gilbert P.B. (2012). Nomenclature for immune correlates of protection after vaccination. Clin Infect Dis.

[22] Andrews N.J., Waight P.A., Burbidge P., Pearce E., Roalfe L., Zancolli M. (2014). Serotype-specific effectiveness and correlates of protection for the 13-valent pneumococcal conjugate vaccine: a postlicensure indirect cohort study. Lancet Infect Dis.

[23] Savulescu C., Krizova P., Valentiner-Branth P., Ladhani S., Rinta-Kokko H., Levy C. (2022). Effectiveness of 10 and 13-valent pneumococcal conjugate vaccines against invasive pneumococcal disease in European children: spidnet observational multicentre study. Vaccine.

[24] Earl P.L., Americo J.L., Wyatt L.S., Espenshade O., Bassler J., Gong K. (2008). Rapid protection in a monkeypox model by a single injection of a replication-deficient vaccinia virus. Proc Natl Acad Sci U S A.

[25] Vollmar J., Arndtz N., Eckl K.M., Thomsen T., Petzold B., Mateo L. (2006). Safety and immunogenicity of IMVAMUNE, a promising candidate as a third generation smallpox vaccine. Vaccine.

[26] von Krempelhuber A., Vollmar J., Pokorny R., Rapp P., Wulff N., Petzold B. (2010). A randomized, double-blind, dose-finding Phase II study to evaluate immunogenicity and safety of the third generation smallpox vaccine candidate IMVAMUNE. Vaccine.

[27] von Sonnenburg F., Perona P., Darsow U., Ring J., von Krempelhuber A., Vollmar J. (2014). Safety and immunogenicity of modified vaccinia Ankara as a smallpox vaccine in people with atopic dermatitis. Vaccine.

[28] Frey S.E., Newman F.K., Kennedy J.S., Sobek V., Ennis F.A., Hill H. (2007). Clinical and immunologic responses to multiple doses of IMVAMUNE (Modified vaccinia Ankara) followed by Dryvax challenge. Vaccine.

[29] Greenberg R.N., Overton E.T., Haas D.W., Frank I., Goldman M., von Krempelhuber A. (2013). Safety, immunogenicity, and surrogate markers of clinical efficacy for modified vaccinia Ankara as a smallpox vaccine in HIV-infected subjects. J Infect Dis.

[30] Ilchmann H., Samy N., Reichhardt D., Schmidt D., Powell J.D., Meyer T.P.H. (2023). One- and two-dose vaccinations with modified vaccinia Ankara-Bavarian nordic induce durable B-Cell memory responses comparable to replicating smallpox vaccines. J Infect Dis.

[31] Overton E.T., Stapleton J., Frank I., Hassler S., Goepfert P.A., Barker D. (2015). Safety and immunogenicity of modified vaccinia Ankara-Bavarian nordic smallpox vaccine in vaccinia-naive and experienced human immunodeficiency virus-infected individuals: an open-label, controlled clinical phase II trial. Open Forum Infect Dis.

[32] Greenberg R.N., Hurley M.Y., Dinh D.V., Mraz S., Vera J.G., von Bredow D. (2015). A multicenter, open-label, controlled phase II study to evaluate safety and immunogenicity of MVA smallpox vaccine (IMVAMUNE) in 18-40 year old subjects with diagnosed atopic dermatitis. PLoS One.

[33] Frey S.E., Winokur P.L., Salata R.A., El-Kamary S.S., Turley C.B., Walter E.B. (2013). Safety and immunogenicity of IMVAMUNE® smallpox vaccine using different strategies for a post event scenario. Vaccine.

[34] Walsh S.R., Wilck M.B., Dominguez D.J., Zablowsky E., Bajimaya S., Gagne L.S. (2013). Safety and immunogenicity of modified vaccinia Ankara in hematopoietic stem cell transplant recipients: a randomized, controlled trial. J Infect Dis.

[35] Duffy J., Myers T.R., Marquez P., Rouse D., Brown H., Zhang B. (2024). JYNNEOS vaccine safety surveillance during the 2022 mpox outbreak using the vaccine adverse event reporting system and V-safe, United States, 2022 to 2023. Sex Transm Dis.

[36] Duffy J., Marquez P., Moro P., Weintraub E., Yu Y., Boersma P. (2022). Safety monitoring of JYNNEOS vaccine during the 2022 Mpox outbreak - united States, may 22-October 21, 2022. MMWR Morb Mortal Wkly Rep.

[37] Mason L.M.K., Betancur E., Riera-Montes M., Lienert F., Scheele S. (2024). MVA-BN vaccine effectiveness: a systematic review of real-world evidence in outbreak settings. Vaccine.

[38] Back S., Knox B., Coakley C., Deltour N., Jacquot E., Raad H. (2024). Effectiveness and safety of the MVA-BN vaccine against mpox in At-Risk individuals in the United States (USMVAc). Vaccines (Basel).

[39] Oom A.L., Wilson K.K., Yonatan M., Rettig S., Youn H.A., Tuen M. (2025). The two-dose MVA-BN mpox vaccine induces a nondurable and low avidity MPXV-specific antibody response. J Virol.

[40] Berry M.T., Khan S.R., Schlub T.E., Notaras A., Kunasekaran M., Grulich A.E. (2024). Predicting vaccine effectiveness for mpox. Nat Commun.

[41] Cohn H., Bloom N., Cai G.Y., Clark J.J., Tarke A., Bermúdez-González M.C. (2023). Mpox vaccine and infection-driven human immune signatures: an immunological analysis of an observational study. Lancet Infect Dis.

[42] Borrow R., Balmer P., Miller E. (2005). Meningococcal surrogates of protection--serum bactericidal antibody activity. Vaccine.

[43] Ladhani Shamez N., Andrews N., Parikh Sydel R., Campbell H., White J., Edelstein M. (2020). Vaccination of infants with meningococcal group B vaccine (4CMenB) in England. N Engl J Med.

[44] Cinconze E., Rosillon D., Rappuoli R., Vadivelu K., Bekkat-Berkani R., Abbing-Karahagopian V. (2023). Challenges in synthesis of real-world vaccine effects on meningococcal serogroup B disease for 4CMenB vaccine post-licensure effectiveness studies: a systematic review. Vaccine.

[45] Rid A., Miller F.G. (2016). Ethical rationale for the Ebola "Ring Vaccination" trial design. Am J Public Health.

[46] Henao-Restrepo A.M., Camacho A., Longini I.M., Watson C.H., Edmunds W.J., Egger M. (2017). Efficacy and effectiveness of an rVSV-vectored vaccine in preventing Ebola virus disease: final results from the Guinea ring vaccination, open-label, cluster-randomised trial (Ebola Ça Suffit!). Lancet.

[47] Muyembe J.J., Pan H., Peto R., Diallo A., Touré A., Mbala-Kingebene P. (2024). Ebola outbreak response in the DRC with rVSV-ZEBOV-GP ring vaccination. N Engl J Med.

[48] Burt F.J., Chen W., Miner J.J., Lenschow D.J., Merits A., Schnettler E. (2017). Chikungunya virus: an update on the biology and pathogenesis of this emerging pathogen. Lancet Infect Dis.

[49] Bartholomeeusen K., Daniel M., LaBeaud D.A., Gasque P., Peeling R.W., Stephenson K.E. (2023). Chikungunya fever. Nat Rev Dis Primers.

[50] Rama K., de Roo A.M., Louwsma T., Hofstra H.S., Gurgel do Amaral G.S., Vondeling G.T. (2024). Clinical outcomes of chikungunya: a systematic literature review and meta-analysis. PLoS Neglected Trop Dis.

[51] Morello C.S., Anantha R., Comer J.E., Mendy J., Tindale L.C., Richardson J.S. (2026). Passive transfer of human sera from chikungunya virus virus-like particle vaccine (Vimkunya) recipients fully protects non-human primates from viremia. npj Vaccines.

[52] Richardson J.S., Anderson D.M., Mendy J., Tindale L.C., Muhammad S., Loreth T. (2024). Chikungunya virus VLP vaccine: phase 3 trial in adolescents and adults. medRxiv.

[53] Tindale L.C., Richardson J.S., Anderson D.M., Mendy J., Muhammad S., Loreth T. (2024). Chikungunya virus VLP vaccine: phase 3 trial in adults ≥65 years of age. medRxiv.

[54] Messina J.P., Brady O.J., Scott T.W., Zou C., Pigott D.M., Duda K.A. (2014). Global spread of dengue virus types: mapping the 70 year history. Trends Microbiol.

[55] Gessner B.D., Jiang Q., Van Werkhoven C.H., Sings H.L., Webber C., Scott D. (2019). A public health evaluation of 13-valent pneumococcal conjugate vaccine impact on adult disease outcomes from a randomized clinical trial in the Netherlands. Vaccine.

[56] van Werkhoven C.H., Bolkenbaas M., Huijts S.M., Verheij T.J.M., Bonten M.J.M. (2021). Effects of 13-valent pneumococcal conjugate vaccination of adults on lower respiratory tract infections and antibiotic use in primary care: secondary analysis of a double-blind randomized placebo-controlled study. Clin Microbiol Infect.

[57] Bollaerts K., Fletcher M.A., Suaya J.A., Hanquet G., Baay M., Gessner B.D. (2022). Vaccine-preventable disease incidence based on clinically, radiologically, and etiologically confirmed outcomes: systematic literature review and Re-analysis of pneumococcal conjugate vaccine efficacy trials. Clin Infect Dis.

[58] Andrews N., Stowe J., Kirsebom F., Toffa S., Rickeard T., Gallagher E. (2022). Covid-19 vaccine effectiveness against the omicron (B.1.1.529) variant. N Engl J Med.

[59] Kirsebom F.C.M., Harman K., Lunt R.J., Andrews N., Groves N., Abdul Aziz N. (2023). Vaccine effectiveness against hospitalisation estimated using a test-negative case-control study design, and comparative odds of hospital admission and severe outcomes with COVID-19 sub-lineages BQ.1, CH.1.1. and XBB.1.5 in England. Lancet Reg Health Eur.

[60] Abdul Aziz N., Kirsebom F.C.M., Allen A., Andrews N. (2025). Effectiveness of spring 2024 (XBB.1.5) and autumn 2024 (JN.1) COVID-19 vaccination against hospitalisation in England. Vaccine.

[61] Tartof S.Y., Slezak J.M., Puzniak L., Hong V., Frankland T.B., Xie F. (2023). Effectiveness and durability of BNT162b2 vaccine against hospital and emergency department admissions due to SARS-CoV-2 omicron sub-lineages BA.1 and BA.2 in a large health system in the USA: a test-negative, case-control study. Lancet Respir Med.

[62] Tartof S.Y., Slezak J.M., Puzniak L., Hong V., Frankland T.B., Ackerson B.K. (2022). Effectiveness of a third dose of BNT162b2 mRNA COVID-19 vaccine in a large US health system: a retrospective cohort study. Lancet Reg Health, Am.

[63] Caffrey A.R., Appaneal H.J., Lopes V.V., Puzniak L., Zasowski E.J., Jodar L. (2024). Effectiveness of BNT162b2 XBB vaccine in the US veterans affairs healthcare System. Nat Commun.

[64] Tartof S.Y., Slezak J.M., Puzniak L., Frankland T.B., Ackerson B.K., Jodar L. (2024). Effectiveness of BNT162b2 XBB vaccine against XBB and JN.1 sublineages. Open Forum Infect Dis.

[65] Appaneal H.J., Lopes V.V., Puzniak L., Zasowski E.J., Jodar L., McLaughlin J.M. (2025). Early effectiveness of the BNT162b2 KP.2 vaccine against COVID-19 in the US veterans affairs healthcare system. Nat Commun.

[66] Falsey A.R., Treanor J.J., Tornieporth N., Capellan J., Gorse G.J. (2009). Randomized, double-blind controlled phase 3 trial comparing the immunogenicity of high-dose and standard-dose influenza vaccine in adults 65 years of age and older. J Infect Dis.

[67] DiazGranados C.A., Dunning A.J., Kimmel M., Kirby D., Treanor J., Collins A. (2014). Efficacy of high-dose versus standard-dose influenza vaccine in older adults. N Engl J Med.

[68] Macias A.E., McElhaney J.E., Chaves S.S., Nealon J., Nunes M.C., Samson S.I. (2021). The disease burden of influenza beyond respiratory illness. Vaccine.

[69] Warren-Gash C., Bhaskaran K., Hayward A., Leung G.M., Lo S.V., Wong C.M. (2011). Circulating influenza virus, climatic factors, and acute myocardial infarction: a time series study in England and Wales and Hong Kong. J Infect Dis.

[70] López-Cuadrado T., de Mateo S., Jiménez-Jorge S., Savulescu C., Larrauri A. (2012). Influenza-related mortality in Spain, 1999-2005. Gac Sanit.

[71] Zucs P., Buchholz U., Haas W., Uphoff H. (2005). Influenza associated excess mortality in Germany, 1985-2001. Emerg Themes Epidemiol.

[72] Fröbert O., Götberg M., Erlinge D., Akhtar Z., Christiansen E.H., MacIntyre C.R. (2021). Influenza vaccination after myocardial infarction: a randomized, double-blind, placebo-controlled, multicenter trial. Circulation.

[73] Lee J.K.H., Lam G.K.L., Yin J.K., Loiacono M.M., Samson S.I. (2023). High-dose influenza vaccine in older adults by age and seasonal characteristics: systematic review and meta-analysis update. Vaccine X.

[74] Nealon J., Modin D., Ghosh R.E., Rudin D., Gislason G., Booth H.P. (2022). The feasibility of pragmatic influenza vaccine randomized controlled real-world trials in Denmark and England. NPJ Vaccines.

[75] Hollingsworth R., Palmu A., Pepin S., Dupuy M., Shrestha A., Jokinen J. (2021). Effectiveness of the quadrivalent high-dose influenza vaccine for prevention of cardiovascular and respiratory events in people aged 65 years and above: rationale and design of a real-world pragmatic randomized clinical trial. Am Heart J.

[76] Johansen N.D., Modin D., Loiacono M.M., Harris R.C., Dufournet M., Larsen C.S. (2026). A pragmatic individually randomized trial to evaluate the effectiveness of high-dose vs standard-dose influenza vaccine in older adults: rationale and design of the DANFLU-2 trial. Am Heart J.

[77] Johansen ND, Modin D, Pardo-Seco J, Rodriguez-Tenreiro-Sánchez C, Loiacono MM, Harris RC, et al. High-dose vs. standard-dose influenza vaccine and cardiovascular outcomes in older adults: the FLUNITY-HD prespecified pooled analysis. Circulation.0. 10.1161/CIRCULATIONAHA.125.077801 PMC12991342 41212981

[78] Johansen N.D., Modin D., Pardo-Seco J., Rodriguez-Tenreiro-Sánchez C., Loiacono M.M., Harris R.C. (2025). Effectiveness of high-dose influenza vaccine against hospitalisations in older adults (FLUNITY-HD): an individual-level pooled analysis. Lancet.

[79] Walsh E.E., Pérez Marc G., Zareba A.M., Falsey A.R., Jiang Q., Patton M. (2023). Efficacy and safety of a bivalent RSV prefusion F vaccine in older adults. N Engl J Med.

[80] Walsh E.E., Pérez Marc G., Falsey A.R., Jiang Q., Eiras D., Patton M. (2024). RENOIR trial - RSVpreF vaccine efficacy over two seasons. N Engl J Med.

[81] Walsh E.E., Eiras D., Woodside J., Jiang Q., Patton M., Marc G.P. (2025). Efficacy, immunogenicity, and safety of the bivalent RSV prefusion F (RSVpreF) vaccine in older adults over 2 RSV seasons. Clin Infect Dis.

[82] Tartof S.Y., Aliabadi N., Goodwin G., Slezak J., Hong V., Ackerson B. (2024). Estimated vaccine effectiveness for respiratory syncytial virus-related lower respiratory tract disease. JAMA Netw Open.

[83] Payne A.B., Watts J.A., Mitchell P.K., Dascomb K., Irving S.A., Klein N.P. (2024). Respiratory syncytial virus (RSV) vaccine effectiveness against RSV-associated hospitalisations and emergency department encounters among adults aged 60 years and older in the USA, October, 2023, to March, 2024: a test-negative design analysis. Lancet.

[84] Bajema K.L., Yan L., Li Y., Argraves S., Rajeevan N., Fox A. (2025). Respiratory syncytial virus vaccine effectiveness among US veterans, September, 2023 to March, 2024: a target trial emulation study. Lancet Infect Dis.

[85] Symes R., Whitaker H.J., Ahmad S., Arnold D., Banerjee S., Evans C.M. (2025). Vaccine effectiveness of a bivalent respiratory syncytial virus (RSV) pre-F vaccine against RSV-associated hospitalisation among adults aged 75-79 years in England. medRxiv.

[86] Lassen M.C.H., Johansen N.D., Christensen S.H., Aliabadi N., Skaarup K.G., Modin D. (2025). RSV prefusion F vaccine for prevention of hospitalization in older adults. N Engl J Med.

[87] Lassen M.C.H., Christensen S.H., Johansen N.D., Aliabadi N., Skaarup K.G., Modin D. (2026). A pragmatic individually randomized trial to evaluate bivalent RSV prefusion F protein-based vaccine effectiveness for preventing RSV hospitalizations in adults aged 60 years or above (DAN-RSV): rationale and trial design. Am Heart J.

[88] Skaarup K.G., Lassen M.C.H., Johansen N.D., Christensen S.H., Aliabadi N., Modin D. (2025). Effect of RSV vaccine on heart failure hospitalizations: a prespecified analysis of the DAN-RSV trial. JACC (J Am Coll Cardiol).

[89] Pareek M., Lassen M.C.H., Johansen N.D., Christensen S.H., Aliabadi N., Skaarup K.G. (2025). Effectiveness of bivalent respiratory syncytial virus prefusion F protein-based vaccine in individuals with or without atherosclerotic cardiovascular disease: the DAN-RSV trial. Eur Heart J.

[90] Wedderburn C.J., Bondar J., Lake M.T., Nhapi R., Barnett W., Nicol M.P. (2024). Risk and rates of hospitalisation in young children: a prospective study of a South African birth cohort. PLOS Glob Publ Health.

[91] Madhi S.A., Polack F.P., Piedra P.A., Munoz F.M., Trenholme A.A., Simões E.A.F. (2020). Respiratory syncytial virus vaccination during pregnancy and effects in infants. N Engl J Med.

[92] Dieussaert I., Hyung Kim J., Luik S., Seidl C., Pu W., Stegmann J.U. (2024). RSV prefusion F protein-based maternal vaccine - preterm birth and other outcomes. N Engl J Med.

[93] Palmu A.A., Jokinen J., Nieminen H., Syrjänen R., Ruokokoski E., Puumalainen T. (2014). Vaccine effectiveness of the pneumococcal Haemophilus influenzae protein D conjugate vaccine (PHiD-CV10) against clinically suspected invasive pneumococcal disease: a cluster-randomised trial. Lancet Respir Med.

[94] Palmu A.A., Jokinen J., Nieminen H., Rinta-Kokko H., Ruokokoski E., Puumalainen T. (2018). Vaccine-preventable disease incidence of pneumococcal conjugate vaccine in the Finnish invasive pneumococcal disease vaccine trial. Vaccine.

[95] International Council For Harmonisation Of Technical Requirements For Pharmaceuticals For Human Use (ICH) (2025).

[96] Komura T., Watanabe M., Shioda K. (2025). Exploring the application of target trial emulation in vaccine evaluation: scoping review. Am J Epidemiol.

[97] Wu E., Rogawski McQuade E., Stensrud M., Nabi R., Benkeser D. (2025). Target trial emulation without matching: a more efficient approach for evaluating vaccine effectiveness using observational data. ArXiv.

